# Probable Treatment Targets for Diabetic Retinopathy Based on an Integrated Proteomic and Genomic Analysis

**DOI:** 10.1167/tvst.12.2.8

**Published:** 2023-02-06

**Authors:** Anddre Osmar Valdivia, Ye He, Xinjun Ren, Dejia Wen, Lijie Dong, Hossein Nazari, Xiaorong Li

**Affiliations:** 1 Department of Ophthalmology and Visual Neuroscience, University of Minnesota Medical School, Minneapolis, MN, USA; 2 Tianjin Key Laboratory of Retinal Functions and Diseases, Tianjin Branch of National Clinical Research Center for Ocular Disease, Eye Institute and School of Optometry, Tianjin Medical University Eye Hospital, Tianjin, China

**Keywords:** bioinformatics, diabetic retinopathy, drug discovery, proteomics, genomics, MMP-13, LGALS3, RUNX2

## Abstract

**Purpose:**

Using previously approved medications for new indications can expedite the lengthy and expensive drug development process. We describe a bioinformatics pipeline that integrates genomics and proteomics platforms to identify already-approved drugs that might be useful to treat diabetic retinopathy (DR).

**Methods:**

Proteomics analysis of vitreous humor samples from 12 patients undergoing pars plana vitrectomy for DR and a whole genome dataset (UKBiobank TOPMed-imputed) from 1330 individuals with DR and 395,155 controls were analyzed independently to identify biological pathways associated with DR. Common biological pathways shared between both datasets were further analyzed (STRING and REACTOME analyses) to identify target proteins for probable drug modulation. Curated target proteins were subsequently analyzed by the BindingDB database to identify chemical compounds they interact with. Identified chemical compounds were further curated through the Expasy SwissSimilarity database for already-approved drugs that interact with target proteins.

**Results:**

The pathways in each dataset (proteomics and genomics) converged in the upregulation of a previously unknown pathway involved in DR (RUNX2 signaling; constituents MMP-13 and LGALS3), with an emphasis on its role in angiogenesis and blood–retina barrier. Bioinformatics analysis identified U.S. Food and Drug Administration (FDA)-approved medications (raltitrexed, pemetrexed, glyburide, probenecid, clindamycin hydrochloride, and ticagrelor) that, in theory, may modulate this pathway.

**Conclusions:**

The bioinformatics pipeline described here identifies FDA-approved drugs that can be used for new alternative indications. These theoretical candidate drugs should be validated with experimental studies.

**Translational Relevance:**

Our study suggests possible drugs for DR treatment based on an integrated proteomics and genomics pipeline. This approach can potentially expedite the drug discovery process by identifying already-approved drugs that might be used for new indications.

## Introduction

The International Diabetes Federation estimated that 463 million individuals had diabetes mellitus (DM) worldwide in 2019 and predicted that this number will increase to 700 million by 2045.^1^ Diabetic retinopathy (DR) is the leading cause of blindness in the working population, and the social and economic impacts of the loss of vision due to diabetes are increasing astronomically as the number of individuals with DM increases. DR is currently treated with pharmacological agents, as well as laser and surgical treatments.^2^ However, there remains a fraction of the diabetic population that are poor responders to current medical and surgical treatments and can potentially benefit from novel therapeutics. Despite such an urgent need for new therapies, the process of identifying disease targets and generating therapeutic compounds for those targets is long and expensive.

The integration of computer science into the biological field has created a surge of high-throughput data and has challenged the science community to store, organize, and extract meaningful information from them.^3^ Gene- and protein-interaction predictor databases, chemical structural databases, and biological pathway databases have been created to enable scientists to extract meaning from this vast amount of information. In addition to offering unprecedented insight into the biological process involved in the pathogenesis of diseases, such data extraction tools have revolutionized drug target discovery and implementation with several novel treatments already emerged utilizing them.^4^^–^^10^ For example, in the field of cancer biology, multiple studies have implemented bioinformatics tools with successful drug discovery outcomes, including a study by Marstrand et al.,^11^ which identified potential targets and candidate drugs for acute promyelocytic leukemia using computational analysis of publicly available microarray datasets. Similarly, Lv et al.^12^ used a computational model for identifying drug candidates that were previously approved for other indications (nordihydroguaiaretic acid, vorinostat, and indomethacin) for breast and prostate cancers. Valdivia and Bhattacharya^8^ implemented a similar genetic, proteomic, and lipidomic analysis approach to identify a lysolipid with the potential for the treatment of demyelinating diseases, such as multiple sclerosis and optic neuritis, attesting to the versatility of bioinformatics applications to different scientific fields. These studies have resulted in a significantly more efficient pathway to finding new drugs compared to the classic methods for treatment target identification.

The bioinformatics studies mentioned above have often utilized in silico tools at the genetic, proteomic, or pharmacological levels separately.^4^^–^^6^^,^^13^^–^^21^ For example, genetic studies have mainly focused on identifying gene clusters associated with disease^13^^–^^16^ or connecting gene clusters with biological pathways in disease progression.^17^^,^^18^ Others have analyzed proteomic profiles to identify proteins that might serve as biomarkers for disease screening or serve as treatment targets.^19^^–^^21^ Moreover, isolated pharmacological studies have mainly compared already existing targets for diseases such as diabetes.^4^^–^^6^ However, streamlining an integrated bioinformatics pipeline to identify potential pathologic pathways, their protein constituents, and potential treatment targets in genomic, proteomic, biochemical, and pharmacological datasets remains to be explored in the field of ocular disease.

This study integrated whole genomic sequencing data from the UKBiobank dataset and proteomics data from samples collected from patients with DR who underwent pars plana vitrectomy to identify already-approved drugs that might be repurposed for the treatment of DR. This drug discovery pipeline highlights pathologic pathways shared by the two datasets and identifies probable protein targets within each pathway. In the current report, the two independent proteomics and genomics datasets converged into identical pathways and protein clusters. By applying chemical binding search platforms and pharmacologic agent databases, we identified U.S. Food and Drug Administration (FDA)-approved drugs that, in theory, can be used for the treatment of DR. Such candidate drugs should be confirmed with experimental studies.

## Methods

### Bioinformatics Pipeline

**Figure 1.**
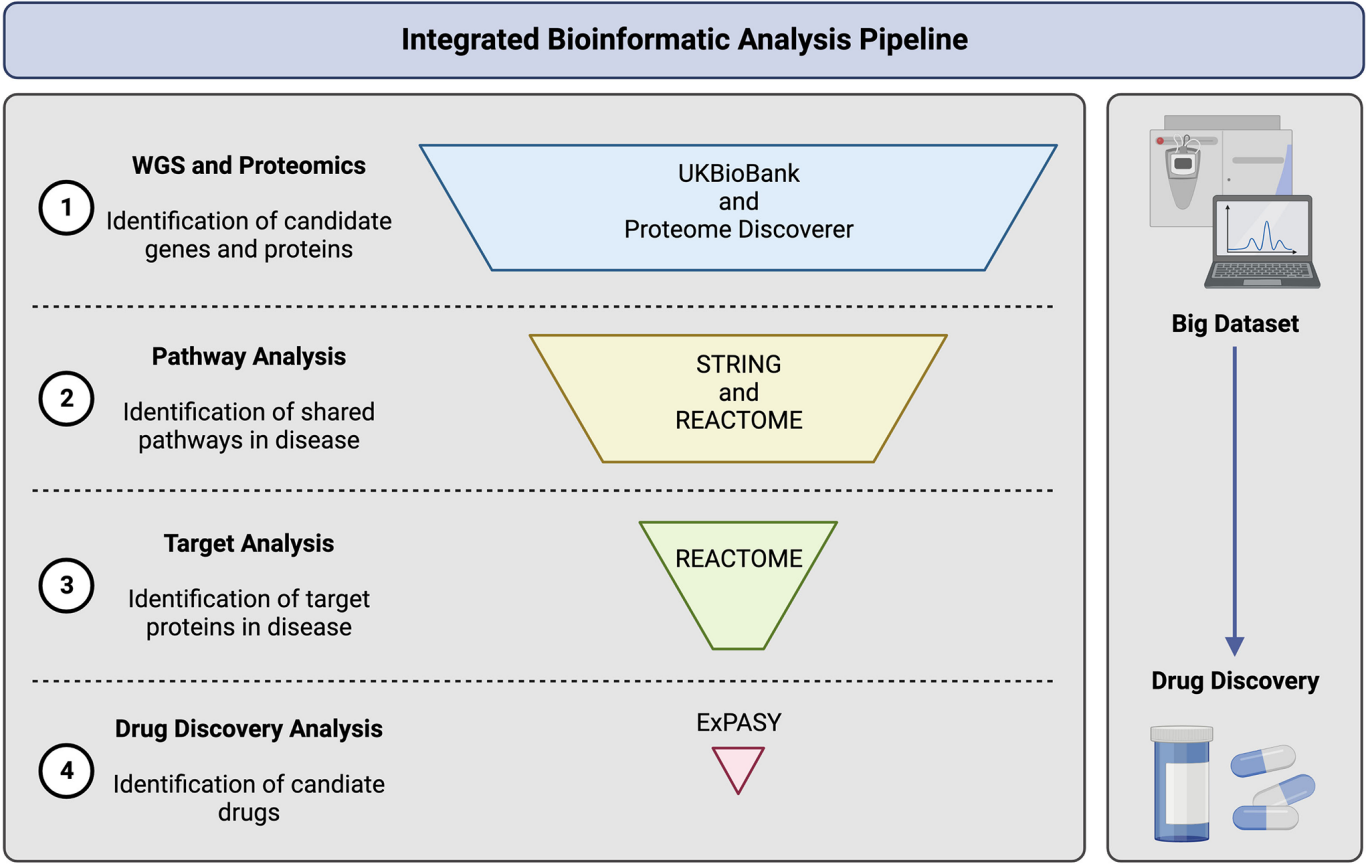
Overview of integrated bioinformatics analysis pipeline. Bioinformatics methodology can be utilized for the identification of potential approved drugs that can modulate the shared pathway between genomic and proteomic datasets. Image was created using BioRender.

**Figure 2.**
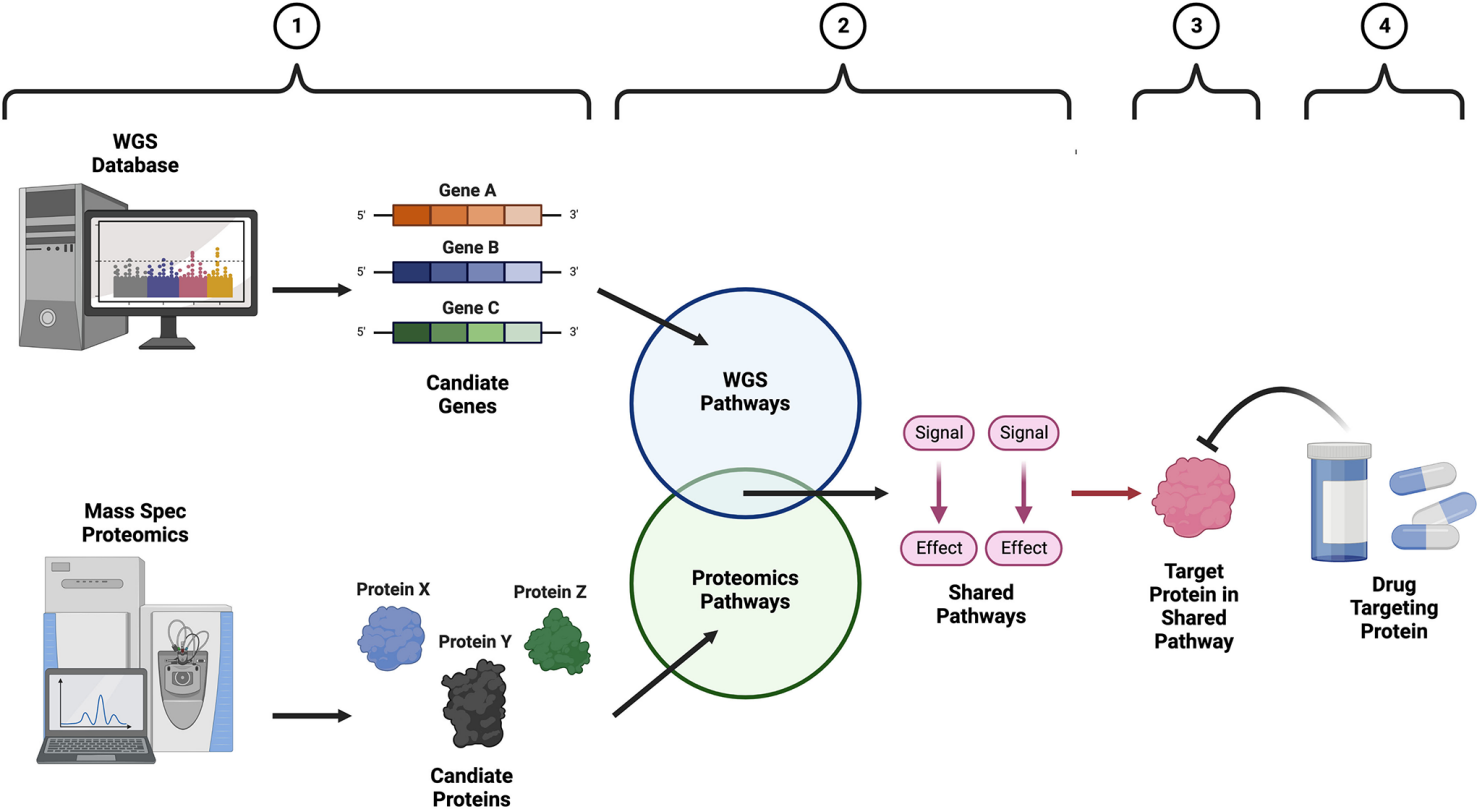
Detailed view of integrated bioinformatics analysis pipeline. In this schematic representation of our integrated bioinformatics analysis, each number located at the top corresponds to the level of analysis numbered in Figure 1. Image was created using BioRender.

The bioinformatics pipeline described here (Figs. 1, 2) integrates four in silico platform levels of analysis that include:

1. Proteomics analysis of human vitreous humor samples from patients with proliferative diabetic retinopathy (PDR) and whole-genome sequencing (WGS) genomic data from the UKBiobank database from patients with DR
2. Identification and comparison of biological pathways in each dataset (proteomic and genomic) to identify shared pathways using the STRING and REACTOME databases
3. Using the REACTOME database to identify potential proteins within the shared pathways to be used as target proteins for drug discovery
4. Using the BindingDB and Expasy SwissSimilarity databases to identify FDA-approved drugs targeting proteins in the shared biological pathway

This bioinformatics pipeline was composed of various publicly available databases (Table 1).

**Table 1.** Public Bioinformatics Platforms Used for the Drug Discovery Pipeline

Database	URL	Brief Description
UKBiobank	https://pheweb.org/UKB-TOPMed/	WGS database with available diseases specific dataset
STRING	https://string-db.org/	Database of known and predicted protein–protein interactions
REACTOME	https://reactome.org/	Open-source, open-access, manually curated and peer-reviewed pathway database
BindingDB	https://www.bindingdb.org/bind/index.jsp	Database for screening experimental based noncovalent association of molecules with known proteins
Expasy SwissSimilarity	http://www.swisssimilarity.ch/	Ligand-based virtual screening tool for rapid screening of libraries of approved drugs, bioactive compounds, and commercially available compounds

### Recruitment of Human Subjects

Patients with diabetic vitreous hemorrhage or tractional retinal detachment due to proliferative DR requiring pars plana vitrectomy were enrolled for vitreous humor sample collection for proteomics analysis. Participants were recruited at the Tianjin Medical University Eye Hospital, Tianjin, China. The research was conducted in compliance with the Health Insurance Portability and Accountability Act and the tenets of the Declaration of Helsinki. Informed consent was obtained from all participants. Twenty-six total vitreous humor samples were collected from 12 patients with proliferative diabetic retinopathy (PDR group). The PDR group did not receive anti-vascular endothelial growth factor (VEGF) treatment or laser treatment at any point within 3 months prior to sample collection. Vitreous samples from 14 patients with epiretinal membranes (ERMs) undergoing pars plana vitrectomy were collected as controls. Individuals with a history of retinal vascular diseases leading to ERM were excluded from the control group.

### Tandem Mass Tag Proteomics

**Figure 3.**
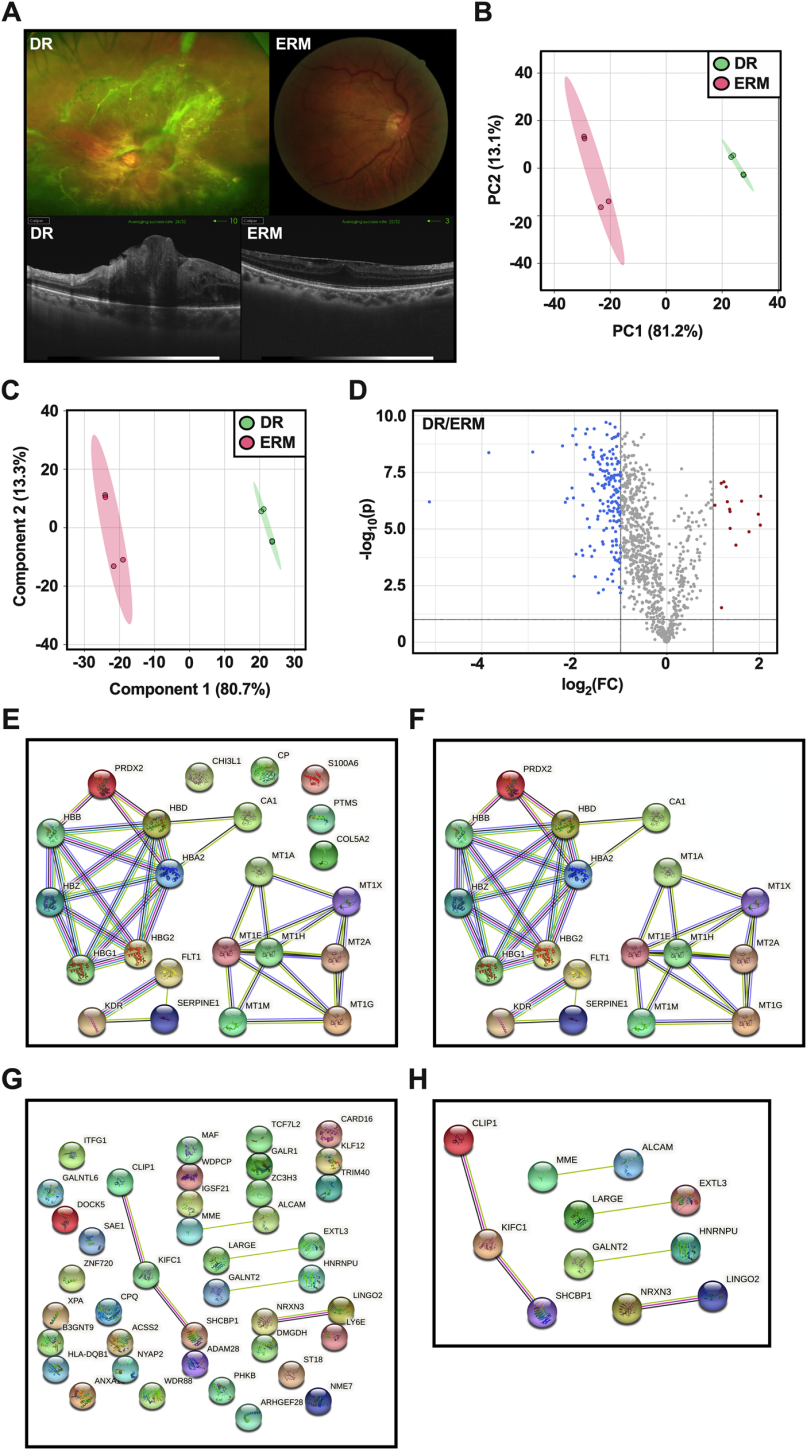
Mass spectrometry proteomics analysis and WGS genomic pathway analysis. (A) Representative color fundus for patients that were included for proteomics analysis. *Top left panel* is a representative ultra-widefield image for proliferative DR. *Top right panel* is a representative color fundus image of the patients recruited with ERMs. The bottom panels are representative optical coherence tomography (OCT) images for each condition. (B) Multivariable dimension reduction analysis (principal component analysis) for proteomics analysis of DR and ERM samples. (C) Bilinear factor model analysis (partial least-squares discriminant analysis) for proteomics analysis of DR and ERM samples. (D) Volcano plot for significant proteins in DR samples compared to ERM samples. *Blue* indicates downregulated proteins in DR; *red*, upregulated proteins in DR. FC threshold = 2; *P* threshold = 0.05; direction of comparison, PDR/ERM. (E) STRING analysis for the interaction of proteomics protein–protein interactions (PPI). PPI *P* < 1.0e-16. (F) Curated STRING analysis showing only proteins that demonstrated an interaction with each other. (G) STRING analysis for the interaction of WGS constituent PPIs. PPI *P* = 0.282. Gene names were utilized as protein names during this analysis. (H) Curated STRING analysis showing only proteins that demonstrated an interaction with each other.

A tandem mass tag (TMT)-based quantitative proteomics strategy was used to screen for differentially expressed proteins of vitreous humor between the DR and ERM groups. The details of TMT method can be found in our previous publications.^22^^,^^23^ In brief, silver-stained blots confirmed decreases in total protein concentrations in the vitreous humor after the depletion of high-abundance proteins relative to those in samples before depletion. Without the interference of high-abundance vitreous proteins, we identified 956 proteins and quantified 853 of these proteins with false discovery rates lower than 0.05% at both the peptide and protein levels. High relevance among these vitreous samples was demonstrated by Pearson correlation coefficients above 0.97. Ninety-seven proteins with altered expression were detected; these included 49 upregulated proteins and 48 downregulated proteins. Peaks were generated using Xcalibur 4.1.31.9 (Thermo Fisher Scientific, Waltham, MA), and proteins were identified using Proteome Discoverer 2.2.0.388. (Thermo Fisher Scientific). Statistical analysis of quantitative proteomics was carried out utilizing MetaboAnalyst 5.0.^24^ Proteins were normalized to sample glyceraldehyde 3-phosphate dehydrogenase (GAPDH). Proteins that were upregulated (*P* < 0.05; fold change [FC] threshold = 2) in the DR group when compared to the ERM group were considered candidates for STRING analysis following the method outlined below (Figs. 3E, 3F).

### Whole-Genome Sequencing Study Datasets

A systematic search of the UKBiobank TOPMed-imputed PheWeb database^25^^,^^26^ was performed using the International Classification of Diseases (ICD) billing code as dictated by the World Health Organization for DR (ICD 250.7). For a detailed description of how the TOPMed whole-genome sequencing (WGS) quality assessment was performed, we refer the reader to the accompanying publications in the TOPMED-imputed PheWeb database.^25^^,^^26^ In brief, a single quality and machine learning pipeline was utilized for all batches, batch effects were analyzed for differences using the genetic principal components, and genotypes were compared with other available genomic databases for consistency. Single predicted loss of function, nonsense, frameshift, and essential splice-site variants were tested for their association with clinical characteristics (either control or disease case) using 1419 PheCodes constructed from the ICD-10 codes as described in a previous publication.^26^ In brief, association analysis of variants to clinical characteristics utilized a logistic mixed model test using the SAIGE (Scalable and Accurate Implementation of GEneralized mixed model) method. The method provides *P* values for case–control ratios and accounts for case–control ratio unbalance, resulting in the output of binary phenotypes (control or disease).^26^ The database included 1330 individuals with DR and 395,155 controls. Single-nucleotide polymorphisms (SNPs) associated with DR were selected based on a minor allelic frequency (MAF) range of 0.000058 < MAF < 0.3 and an effect size (ES) range of 0.24 < ES < 9.3. Candidate variants were curated based on their proximity to the nearest gene, *P* < 0.001, and based on an initial MAF range of 0 to 0.5,^27^ which included both rare (MAF < 0.001) and common (MAF > 0.001) variants, as well as both SNPs and indels, with the addition of inclusion of loss of function and non-synonymous mutations.

### Protein–Protein Interaction Analysis Using the STRING Database

Proteins from the proteomics dataset were analyzed for protein–protein interactions using the STRING database, version 11.5 (minimum required interaction score, 0.4; protein–protein interaction enrichment *P* ≤ 1.0e-16). Only proteins that demonstrated protein–protein interactions (presented as known interactions from experimental evidence and predicted interactions from text miming and co-expression) were considered for pathway analysis following the method outlined below (Figs. 3E, 3F). The corresponding proteins of each gene in the genomic dataset were analyzed for protein–protein interactions using the STRING database (minimum required interaction score, 0.4; protein–protein interaction enrichment *P* = 0.282). Only proteins that demonstrated protein–protein interactions (presented as known interactions from experimental evidence and predicted interactions from text mining and co-expression) were considered for pathway analysis following the method outlined below (Figs. 3G, 3H).

### Identification and Comparison of Proteomic and WGS Pathways

The REACTOME database, version 3.7,^28^^,^^29^ was utilized to analyze candidate pathways associated with genes and proteins identified via WGS and proteomics analysis independently. Parameters applied for the pathway analysis included the “project to human” and “include interactors” features, followed by the curation of top candidate pathways by statistical significance (*P* < 0.05) (Table 2). A side-by-side comparison of the pathways identified for each dataset was curated based on their pathway category (Table 2, column 3), and only the categories shared between the genomic and proteomic datasets were considered for further analysis. Further, a side-by-side comparison of the specific pathways (Table 2, column 4) within each shared category identified the pathways shared between both the genomic and proteomic datasets.

**Table 2.** Pathway Analysis for Proteomics and Genomic Datasets

Dataset	Pathway Identifier	Pathway Category	Pathway Name	Entities *P*
Mass spectrometry (Proteomics)	R-HSA-5661231	Cellular responses to stimuli	Metallothioneins bind metals.	2.21E-13
	R-HSA-195399	Signal transduction	VEGF binds to VEGFR leading to receptor dimerization.	6.91E-04
	R-HSA-6806834	Signal transduction	Signaling by MET	0.001351944
	R-HSA-9673768	Disease associated	Signaling by membrane-tethered fusions of PDGFRA or PDGFRB	0.006833935
	R-HSA-9706369	Immune system response	Negative regulation of FLT3	0.01175446
	R-HSA-5619115	Disease associated	Disorders of transmembrane transporters	0.016165492
	R-HSA-8941333	Gene expression transduction	RUNX2 regulates genes involved in differentiation of myeloid cells.	0.031066791
Whole-genome sequencing (Genomics)	R-HSA-5083627	Disease associated	Defective *LARGE* causes MDDGA6 and MDDGB6.	0.003533237
	R-HSA-6794361	Neuronal system	Neurexins and neuroligins	0.005939488
	R-HSA-5173105	Metabolism of proteins	*O*-linked glycosylation	0.007319633
	R-HSA-6794362	Neuronal system	Protein–protein interactions at synapses	0.014511731
	R-HSA-8856688	Vesicle-mediated transport	Golgi-to-ER retrograde transport	0.019718755
	R-HSA-428359	Metabolism of RNA	Insulin-like growth factor-2 mRNA binding proteins (IGF2BPs/IMPs/VICKZs) bind RNA.	0.020155209
	R-HSA-8941333	Gene expression transduction	RUNX2 regulates genes involved in differentiation of myeloid cells.	0.021022733
	R-HSA-5626467	Signal transduction	RHO GTPases activate IQGAPs.	0.038221149
	R-HSA-1300645	Reproduction	Acrosome reaction and sperm: oocyte membrane binding	0.039073511
	R-HSA-3134963	Immune system response	DEx/H-box helicases activate type I IFN and inflammatory cytokine production.	0.040776089

Pathway analysis of candidate proteins in the mass spectrometry proteomics and WGS genomics datasets. Pathway analysis was performed utilizing the REACTOME database. The “project to human” and “includes interactors” features were included as part of the parameters. Pathways were curated based on a *P* < 0.05. VEGF, vascular endothelial growth factor; VEGFR, vascular endothelial growth factor receptor; MET, hepatocyte growth factor receptor; PDGFRA, platelet-derived growth factor receptor alpha; PDGFRB, platelet-derived growth factor receptor beta; FLT3, FMS-like tyrosine kinase-3; RUNX2, runt-related transcription factor 2; MDDGA6/MDDGB6, congenital muscular dystrophy-dystroglycanopathy with brain and eye anomalies (type A6/B6); ER, endoplasmic reticulum; IGF2BPs/IMPs/VICKZs, insulin-like growth factor 2 mRNA-binding proteins; IQGAPs, IQ motif-containing GTPase-activating proteins; IFN, interferon

#### Identification of Pathway Constituents

The pathway shared between the genomic and proteomic datasets was curated for proteins that converged in the modulation of the expression and activation of the shared pathway, as well as extracellular signals that converged or diverged with the shared pathway (Table 3).

**Table 3.** Target Proteins in the RUNX2 Pathway

Relationship to RUNX2 Pathway	Candidates	UniProtKB	Name
Convergence or divergence with RUNX2 signaling	RUNX2	Q13950	Runt-related transcription factor 2
	MMP-13	P45452	Matrix metalloproteinase 13
	BMP2	P12643	Bone morphogenetic protein 2
	LGALS3	P17931	Galectin-3
	UCMA	Q8WVF2	Unique cartilage matrix-associated protein
Expression and activation of RUNX2	TWIST1	Q15672	Twist-related protein 1
	ESRRA	P11474	Steroid hormone receptor ERR1
	PPARGC1	Q9UBK2	Peroxisome proliferator-activated receptor gamma coactivator 1-alpha
	ESR1	P03372	Estrogen receptor
	CBFβ	Q13951	Core-binding factor subunit beta
	NR3C1	P04150	Glucocorticoid receptor
	NKX3-2	P78367	Homeobox protein Nkx-3.2
	MSX-2	P35548	Homeobox protein MSX-2
	DLX-5	P56178	Homeobox protein DLX-5

Candidate target proteins found in the RUNX2 pathway. Proteins were selected based on their appearance in the RUNX2 pathway using the REACTOME database. Convergent and divergent proteins are extracellular and cytoplasmic proteins in the RUNX2 pathway, apart from RUNX2, which localized to the nucleus. Proteins that were associated with expression and activation of RUNX2 were localized to the nucleus.

#### Identification of Approved Drugs Targeting Proteins in the Shared Pathway

Proteins in the shared pathway were analyzed utilizing the BindingDB database,^30^ searching for chemical compounds that bind to the target protein based on the UniProtKB accession number of the protein. Chemical compounds were curated based on their half-maximal inhibitory concentration (IC_50_). Chemical structures for each compound were further analyzed utilizing the Expasy SwissSimilarity database^31^ to identify approved drugs based on their two-dimensional and three-dimensional stereochemistry (ChemBL-approved drug database). Approved drugs had completed clinical trial phase 4 (Table 4).

**Table 4.** Chemical Compounds Targeting Protein in the RUNX2 Pathway and Their Approved Drugs

Candidates	UniProtKB	BDBM Accession Number	IC_50_ Range (nM)	Drug Name (Clinical Trial Phase 4)
RUNX2	Q13950	Not available	Not available	Not available
MMP-13^a^	P45452	BDBM50025190, BDBM50203953, BDBM50102750, BDBM50025188, BDBM50389100, BDBM50025160, BDBM50025192, BDBM12074, BDBM11863	0.0022–0.01	RaltitrexedPemetrexedGlyburideProbenecid
BMP2	P12643	BDBM50017345, BDBM50017346, BDBM50017347	Not available	Clindamycin palmitate
LGALS3^a^	P17931	BDBM509535, BDBM509530, BDBM509541, BDBM509546, BDBM509536, BDBM509540, BDBM509556, BDBM509542, BDBM509538, BDBM509539	43–86	Clindamycin hydrochlorideTicagrelor
UCMA	Q8WVF2	Not available	Not available	Not available
TWIST1	Q15672	Not available	Not available	Not available
ESRRA	P11474	BDBM50169743, BDBM50212166, BDBM17292, BDBM50212173, BDBM50501982, BDBM50501984, BDBM50171724, BDBM50171726, BDBM50501969, BDBM50276796	2–5	PioglitazoneRosiglitazoneFulvestrantFluoxetine hydrochlorideBisacodylSilodosin
PPARGC1	Q9UBK2	Not available	Not available	Not available
ESR1	P03372	BDBM50276884, BDBM17292, BDBM50523039, BDBM50532101, BDBM368199, BDBM50523049, BDBM50542086	<0.00200–0.031	Raloxifene hydrochlorideToremifene citrateBazedoxifene
CBFβ	Q13951	BDBM123672, BDBM123674, BDBM123675, BDBM123665, BDBM123684, BDBM123673, BDBM123671, BDBM123670, BDBM123659, BDBM123669	700–1400	Abametapir
NR3C1	P04150	BDBM18627, BDBM50250114, BDBM50250125, BDBM50206900, BDBM50203432, BDBM50250115, BDBM141378, BDBM50354851, BDBM50203441, BDBM50203418	0.008–0.04	Fluocinolone acetonideFluorometholoneLoteprednolMedrysoneMethylprednisolonePrednisoloneRimexolone
NKX3-2	P78367	Not available	Not available	Not available
MSX2	P35548	Not available	Not available	Not available
DLX5	P56178	Not available	Not available	Not available

Top 10 compounds that target constituents in the RUNX2 pathway were selected based on their half-maximal inhibitory concentration (IC_50_). Compounds were further analyzed utilizing the Expasy SwissSimilarity database for ChemBL-approved drugs. Approved drugs had a completed clinical trial phase 4 at the time of screening.

a Drugs targeting MMP-13 and LGALS3 have the greatest potential for the treatment of DR.

### Validation of Results Using Literature Search

Proteins that were part of the shared pathway (Table 3) and drugs that target proteins in the shared pathways (Table 4) were subsequently searched in the PubMed database for English-language articles published between the years 2000 and 2022 that were associated with DR (Table 5). The following format was implemented in this search “[Candidate protein/pathway name from Table 3] diabetic retinopathy”, “[Drug name from Table 4] diabetic retinopathy,” and “[Drug name from Table 4] diabetes mellitus.” The search was performed in February 2022 and June 2022.

**Table 5.** Literature Validation of Target Proteins and Drugs Associated With DR

Protein	PMID Numbers for Articles Associating Protein With DR	Drug Targeting Protein (Disease Association: PMID numbers)
RUNX2	29029615, 19383984, 28462510, 32319515, 31830266	Not available
MMP-13	24392031, 28662829, 29853773, 30215334, 22519597, 27454437, 20479714, 24894401, 26599598, 23833698, 20220057	Raltitrexed (cancer)Pemetrexed (cancer/DM: 34439918)Glyburide (DM, DR: 34371786, 33080394)Probenecid (DR: 34690760, 11803477)
BMP2	24910902, 33919531, 33584642, 29046696, 33584642, 32259084, 32707711	Clindamycin palmitate (antibiotic)
LGALS3	21249158, 20490454, 21111733, 34426510, 18554398, 34405947	Clindamycin hydrochloride (antibiotic)Ticagrelor (DM: 30788847, 34923021, 35321823, 32303164)
UCMA	Not available	Not available
TWIST1	25414194, 32234721, 34461141, 34845879, 24600510	Not available
ESRRA	31460821, 28000229, 32210913, 23335958	Pioglitazone (DM: 19183764, 17054272, 30706731, 33995271)
		Rosiglitazone (DR: 18541841, 14598030, 33417950, 31227789, 21599458; DM: 18407718)
		Fulvestrant (cancer)
		Fluoxetine hydrochloride (DM: 10834419, 15847950, 14977450, 21829436)
		Bisacodyl (constipation)
		Silodosin (benign prostatic hyperplasia)
PPARGC1	15782399, 23745551, 21911054, 24703492, 30665952, 16978372, 29436254	Not available
ESR1	35114215, 30787185, 23276812, 35024051, 24093675, 18305958, 31293826, 17327435, 17325140	Raloxifene hydrochloride (DR: 21413859; DM: 17451421, 17308373)Toremifene citrate (cancer)
		Bazedoxifene (DM: 26345606)
		
CBFβ	34173806, 10924741	Abametapir (head lice infection)
NR3C1	31734719, 32017889, 33957209	Fluocinolone acetonide (DR: 20939799, 28081945, 22727177, 31100106)
		Fluorometholone (DR: 35264022, 30549478, 28778572, 28252875)
		Loteprednol (DR: 27293138)
		Medrysone (inflammatory eye diseases)
		Methylprednisolone (DM: 16595011, 15579988)
		Prednisolone (DM: 15180141, 17188065, 28778572)
		Rimexolone (inflammatory eye diseases)
NKX3-2	Not available	Not available
MSX2	26605646, 21193740, 28062508, 17720944, 28202017	Not available
DLX5	12488363, 25913633	Not available

Literature validation for the correlation of target proteins with DR and literature support for the use of drugs in DR and/or DM. Drugs that were used for other disease were listed without specific citations.

## Results

### Proteomic and Genomic Constituents for Pathway Analysis

Vitreous samples taken from human subjects diagnosed with PDR were compared with samples from human subjects diagnosed with ERMs (Fig. 3A).^32^^,^^33^ To assess sample variance a multivariable dimension reduction analysis (principal component analysis) was implemented. Principal component analysis analysis demonstrated no overlap in the 95% confidence region of each group, indicating that each group had a unique proteomic profile (Fig. 3B). Further analysis implementing a bilinear factor model analysis (partial least-squares discriminant analysis [PLS-DA]) demonstrated sample separation and validated the unique proteomic composition of the DR and control groups (Fig. 3C). Significance analysis revealed 25 unique proteins (Fig. 3D, red dots) that were increased in the DR group when compared to the ERM group (Fig. 3D). These proteins were utilized for STRING analysis (Figs. 3E, 3F). Only 18 proteins demonstrated interactions and were subsequently used for pathway analysis. On the other hand, WGS genomic analysis revealed 41 total variants associated with DR (Supplementary Table S1). After variants were analyzed utilizing the protein–protein interaction STRING database, only 11 proteins demonstrated interactions with each other (Figs. 3G, 3H). These proteins were utilized as constituents for pathway analysis.

### Biological Pathway Analysis

Constituents from the proteomics and genomics datasets were independently analyzed utilizing the REACTOME database, which revealed seven biological pathways associated with the proteomics dataset and 10 biological pathways associated with the genomics dataset (Table 2). Major biological pathways associated with the proteomics dataset included pathways associated with cellular response to stimuli, signal transduction, immune system response, disease process, and changes in gene expression. Major biological pathways associated with the genomics dataset included pathways associated with neuronal system response, metabolism of proteins, membrane trafficking, RNA metabolism, gene expression change, signal transduction, reproduction, and immune system response. From this range of biological pathways, only the runt-related transcription factor 2 (RUNX2) pathway (changes in gene expression) was shared between both datasets.

### Identification of Approved Drugs Targeting Proteins in the RUNX2 Pathway

The RUNX2 pathway contains many different constituents that either regulate the expression and activation of RUNX2 or converge/diverge with RUNX2 signaling. Proteins that regulate the expression and activation of RUNX2 include transcriptional regulators, cell receptors, and transcription factors, whereas proteins that converge/diverge with RUNX2 signaling include transcription factors, metalloproteinases, and growth factors (Table 3). Proteins from Table 3 were utilized as query proteins to find chemical compounds that interact with them based on their IC_50_ (Table 4). Only seven proteins were found to have chemical compounds that were predicted by the BindingDB database to have an interaction. Further analysis of the chemical structure and stereochemistry of these compounds utilizing the Expasy SwissSimilarity database found already approved drugs (clinical trial phase 4) with similar chemical properties. The most noteworthy were drugs that target matrix metalloproteinase-13 (MMP-13) (raltitrexed, pemetrexed, glyburide, and probenecid) and galectin-3 (LGALS3) (clindamycin hydrochloride and ticagrelor).

### Literature Validation of Bioinformatics Pipeline

Each protein in the RUNX2 pathway (Table 3) was manually curated for its association with DR in primary research and review articles. The initial retrieval search resulted in 66 primary research and review articles; only UCMA and NKX3-2 were not found to be in association with DR (Table 5). Literature retrieval for drugs associated with DR or DM resulted in 40 primary research and review articles, validating the findings of our integrated bioinformatics approach (Table 5).

## Discussion

The treatment of DR in poor responders to anti-VEGF therapy presents a clinical challenge that may require novel therapeutic drugs aiming at new pathologic targets. Identifying new targets and generating new drugs is long and expensive; however, our proposed integrated bioinformatics analysis pipeline may be able to expedite this process efficiently. We analyzed PDR mass spectrometry proteomics datasets and WGS genomic datasets for the shared biological pathways between both datasets. Interestingly, both of these diverse datasets converged independently in the RUNX2 pathway (Table 2), providing affirmation of the potential contribution of this pathway to DR. Further analysis within the RUNX2 pathway identified MMP-13 and LGALS3 as the most prominent targets for drug modulation (Table 3). Approved drugs that target MMP-13 include raltitrexed, pemetrexed, glyburide, and probenecid, and drugs that target LGALS3 include clindamycin hydrochloride and ticagrelor (Table 4). Some of these drugs have mainly been approved for use as cancer therapies due to their anti-angiogenic properties (Table 5); however, their potential for use in DR remains to be explored. Of note, glyburide is a medication that is used systemically to control blood glucose levels. Although a potential beneficial effect in the retina might be attributed to hypoglycemic effects of glyburide, our study suggests a favorable effect of glyburide in the pathogenesis of DR. This favorable localized effect was also suggested by Berdugo et al.,^34^^,^^35^ who explored the effects of oral and intravitreal glyburide at a non-hypoglycemic dose in animal models of DR and reported reduced DR in treatment groups. Our study proposes mechanistic explanations for such beneficial effects. Further experimental studies should validate the possible beneficial effects of the above medication in DR, as it is “theoretically” suggested by our approach. However, our findings, although theoretical, are biologically plausible as detailed in the next few paragraphs.

RUNX2 belongs to a family of RUNX transcription factors (RUNX1, RUNX2, and RUNX3) with a wide range of biological functions, including embryonic development, cell proliferation, differentiation, lineage determination, apoptosis, and hematopoiesis.^36^ To properly function as transcription factors, RUNX factors require the formation of a heterodimer with core binding factor β (CBFβ) (Table 3), which is ubiquitously expressed in RUNX-expressing cells.^36^^–^^39^ In our study, proteins that were upregulated in DR converge in the RUNX2 pathway activation, which is supported by a recent study that reported RUNX2 upregulation in DR and its role in the breakdown of the blood–retinal barrier (BRB).^40^ It is noteworthy to mention that previous studies looking at the RUNX2 pathway have reported a downregulation of the RUNX2 pathway in diabetes^41^^–^^43^; however these studies primarily focused on bone formation, osteogenesis, and regulation of bone development,^41^^–^^47^ whereas our study identified upregulation of RUNX2 in myeloid cell differentiation. Myeloid cells (monocytes and macrophages) are an important component in the pathology of DR, where changes in the myeloid cell compartment have been reported to be altered in diabetes.^48^ Of particular interest, monocyte activation has been associated with degradation of the BRB and progression of DR into PDR, supporting our findings regarding the contribution of RUNX2 in DR pathology through myeloid cells.^49^

LGALS3 is a β-galactoside-binding protein that has been associated with various biological functions that include cell–cell adhesion, cell–matrix interactions, macrophage activation, angiogenesis, metastasis, and apoptosis.^50^ As an angiogenic factor, LGALS3 has been documented to be upregulated in DR,^51^ to enhance the proliferation and angiogenesis of endothelial cells,^52^ and to promote vascularization of cancers.^53^ Despite its heterogeneous function as an angiogenic factor, LGALS3 requires cleavage by MMP to promote angiogenesis.^54^^,^^55^ Our bioinformatics approach identified the upregulation of MMP-13 (LGALS3 is a substrate for MMP-13^56^^,^^57^) and the metallothioneins pathway (important in the expression of metalloproteinases^58^), providing not only upregulation of an angiogenic factor (LGALS3) but also a means for activating its angiogenic properties (Tables 2, 3).

Furthermore, capillary nonperfusion and ischemic events underlie the progression of DR into PDR which is driven by hypoxia and the production of factors that promote neovascularization.^59^ Remodeling of vasculature during neovascularization and alterations of the BRB are events associated with DR and have been implicated with metalloproteinase activity.^60^ Disruption to the BRB has been associated with MMP-2, MMP-9, and MMP-14,^60^ and neovascularization has been associated with MMP-2 and MMP-9.^60^ A study looking into changes in the vasculature microenvironment found that, after 3 days of ischemia, MMP-2, MMP-3, and MMP-13 levels were elevated in diabetic mice compared to non-diabetics.^61^ However, elevated levels of MMP-2, MMP-3, and MMP-13 did not increase collagenolysis and vascular remodeling, pointing toward MMP-13 contributing to DR through other means. A different study demonstrated that elevated levels of MMP-13 and inflammatory markers in human monocytes were associated with hyperglycemic conditions, suggesting that MMP-13 might contribute to DR through its action in myeloid cells.^62^ This notion is supported by the known association of activated monocytes with progression of DR into PDR, the degradation of the BRB,^49^^,^^63^^–^^65^ and our findings of upregulation of RUNX2 in myeloid cells. In addition, LGALS3 has been associated with *O*-glycosylation in corneal epithelial cells^66^ and in the maturation of cancers, including vascularization and metastasis.^67^^,^^68^ This is another crucial aspect of the role of LGALS3, as our bioinformatics approach also identified the upregulation of *O*-linked glycosylation (Table 2).

In their publication, Berdugo et al.^34^ demonstrated that glyburide exerts its retinal protective effects through the inhibition of sulfonylurea receptor 1 (SUR1). SUR1 has been documented to propagate the pathological effects induced by hypoxia and ischemia through the promotion of neuroinflammation and disruption of the BRB via MMP-9.^60^^,^^69^ Furthermore, glyburide has been documented to have direct inhibitory effects on many metalloproteinases, including MMP-13.^70^ Therefore, the retinal protective effects of glyburide in DR might be a combination of inhibition of SUR1 and MMP-13, which will require further exploration.

The identification of these molecular events resulted from the independent conversion of two separate datasets (genomic and proteomic) pointing toward RUNX2 pathway activation being associated with DR, as recently demonstrated by an experimental study.^40^ Within this pathway, MMP-13 and LGALS3 became essential target proteins as they have been associated with DR. Therefore, the drugs identified by our bioinformatics approach raise the possibility that they might have beneficial effects in the treatment of DR. However, their utility and efficacy for this purpose remain to be explored, with efforts already being undertaken for the direct effect of glyburide in decreasing DR.^34^^,^^35^ Like glyburide, the other medications identified by our bioinformatics approach that target MMP-13 might have similar effects in the retina due to their similarity in chemical structure. Furthermore, our literature search identified several medications that have been investigated for their potential effects on DR or DM, supporting our objective bioinformatics approach (Table 5).

The results of our study should be inspected considering its theoretical nature and the limitations and strength of this approach. We explored shared pathways between proteomics of “advanced DR” from an Asian population and genomics of “any DR” from a mostly European population (the UKBiobank does not identify DR subclasses, so individuals with early and advanced DR are all included in one category^71^). It should be noted that this is not a comparative study; thus, the genotypical and phenotypical diversity of the two groups does not invalidate the results. On the contrary, the diversity of these two databases representing “advanced DR” from an Asian population and “any DR” from a mostly European population adds to the credibility of its finding. In addition, even though genomics data from individuals with early DR are included in this study, the biologic pathways leading to DR start many years before the clinical presentation and progression of the disease^72^; thus, it is conceivable that even individuals with early DR (lumped together with participants with advanced DR in the UKBiobank) have activated pathways leading to more advanced stages of DR. Finally, despite the fact that our proteomics and genomics data are from populations with limited ethnic diversity, the association of ethnic genetic constructs with DR development and progression is unclear.^73^

Our study is a theoretical venture and the candidate drugs identified by its approach must be validated in experimental studies. The outcomes of the pipeline we suggest depend on when the search is run. The REACTOME and other pathway databases such as PANTHER and KEGG are a consortium of various public academic institutions that gradually and manually review, curate, and include newly found pathways in the database. As new pathways are being discovered they will be included in the database with a lag. Thus, the pathways observed in our study are reflective of the status of the REACTOME database at the time of our analysis, which is expected to be updated in future.

We analyzed proteomics and genomics information from two diverse population with extended analyses to include a drug discovery arm. Each step of the pipeline provided input for the subsequent dataset, eventually converging at a shared target that can in theory be potentially modified with available drugs. Doing so, our approach can accelerate drug discovery and significantly reduce the costs of finding new therapeutics. In particular, identifying already FDA-approved drugs largely expedites their application for bedside treatment. Previous studies (mostly in the field of cancer medicine^74^^–^^77^) have applied this tool in isolation or missed the connection between genomics and proteomics data and drug discovery search engines. It is clear that the application of our bioinformatics pipeline extends beyond DR and ocular diseases and can be implemented in a wide range of medical fields. In short, the approach used in this pipeline is robust, and the method of finding approved drugs to be repurposed for new indications is sound and efficient. Our approach stands to bridge the bench-to-bedside gap that is often a rate-limiting step in developing novel treatments for the management of DR after their efficacy has been further validated in preclinical and clinical studies.

## Supplementary Material

Supplement 1
