# Supplementary material for: Probable Treatment Targets for Diabetic Retinopathy Based on an Integrated Proteomic and Genomic Analysis
Source: Transl Vis Sci Technol. 2023 Feb 6;12(2):8. doi: 10.1167/tvst.12.2.8 (PMC9910385; doi:10.1167/tvst.12.2.8)
Supplement: Supplement 1 [file tvst-12-2-8_s001.docx]

**Full Title:** Probable treatment targets for diabetic retinopathy based on an integrated proteomic and genomic analysis

**Authors:** Anddre Osmar Valdivia^1,2,*^, Ye He^3,*^, Xinjun Ren^3^, Dejia Wen^3^, Lijie Dong^3^, Hossein Nazari^1,2,†^, and Xiaorong Li^3,†^.

1. Department of Ophthalmology and Visual Neuroscience, University of Minnesota, Minneapolis, MN 55455, United States.

2. University of Minnesota Medical School, Minneapolis, MN 55455, United States.

3. Tianjin Key Laboratory of Retinal Functions and Diseases, Tianjin Branch of National Clinical Research Center for Ocular Disease, Eye Institute and School of Optometry, Tianjin Medical University Eye Hospital, Tianjin, China

*** Co-first author:** Anddre Osmar Valdivia and Ye He contributed equally to this work.

**† Corresponding authors:** Xiaorong Li and Hossein Nazari.

**Supplemental Table:**

| **Supplemental Table 1. Top loci genetic variations associated with DR, based on UKBiobank database** | | | | |
| --- | --- | --- | --- | --- |
| **Variant** | **Nearest Gene(s)** | **MAF (C/R)** | **P-value** | **Effect Size (se)** |
| 6:32,658,698 G / A (rs9273368) | HLA-DQB1 | 0.3 (C) | 2.40E-40 | 0.59 (0.044) |
| 10:112,998,590 C / T (rs7903146) | TCF7L2 | 0.29 (C) | 6.70E-10 | 0.27 (0.043) |
| 16:31,750,081 C / T (rs1008012616) | ZNF720 | 0.00019 (R) | 6.50E-08 | 21 (3.9) |
| 9:97,677,438 C / T (rs62560504) | XPA | 0.0039 (C) | 1.00E-07 | 2.0 (0.38) |
| 8:143,472,984 C / T (rs372047048) | ZC3H3 | 0.00033 (R) | 1.30E-07 | 14 (2.6) |
| 9:29,221,949 C / T (rs1464865423) | LINGO2 | 0.000058 (R) | 1.40E-07 | 30 (5.8) |
| 9:29,894,305 T / C (rs966764262) | LINGO2 | 0.000062 (R) | 1.50E-07 | 30 (5.7) |
| 8:25,079,474 T / A (rs75761903) | DOCK5 | 0.017 (C) | 1.70E-07 | 0.87 (0.17) |
| 2:226,059,280 GT / G (rs200308611) | NYAP2 | 0.012 (C) | 1.90E-07 | 1.0 (0.19) |
| 19:33,150,850 C / T (rs10423969) | WDR88 | 0.24 (C) | 1.90E-07 | 0.24 (0.046) |
| 8:96,718,759 C / A (rs759772534) | CPQ | 0.0013 (C) | 1.90E-07 | 3.9 (0.75) |
| 22:33,446,278 T / C (rs80332473) | LARGE1 | 0.072 (C) | 2.00E-07 | 0.40 (0.078) |
| 6:33,387,741 C / T (rs115337486) | KIFC1 | 0.026 (C) | 2.10E-07 | 0.68 (0.13) |
| 2:63,532,779 T / A (rs904921015) | WDPCP | 0.00011 (R) | 3.90E-07 | 24 (4.8) |
| 1:244,916,088 CA / C (rs779387238) | HNRNPU | 0.0018 (C) | 4.20E-07 | 3.4 (0.67) |
| 3:105,575,078 T / C (rs753888375) | ALCAM | 0.00023 (R) | 4.30E-07 | 12 (2.3) |
| 6:30,126,336 C / G (rs2844790) | TRIM40 | 0.15 (C) | 4.60E-07 | 0.28 (0.055) |
| 16:79,699,594 G / A (rs138735020) | MAF | 0.0003 (R) | 5.20E-07 | 9.3 (1.9) |
| 1:17,997,628 G / A (rs565963730) | IGSF21 | 0.00095 (R) | 5.50E-07 | 4.6 (0.92) |
| 8:52,469,658 C / T (rs772456076) | ST18 | 0.00023 (R) | 5.50E-07 | 12 (2.3) |
| 14:78,851,624 G / A (rs758902011) | NRXN3 | 0.000088 (R) | 6.00E-07 | 23 (4.7) |
| 12:122,357,999 G / C (rs55639239) | CLIP1 | 0.0072 (C) | 6.10E-07 | 1.3 (0.26) |
| 8:28,713,613 C / T (rs972181831) | EXTL3 | 0.000067 (R) | 6.10E-07 | 44 (8.8) |
| 8:24,167,614 A / G (rs748661592) | ADAM28 | 0.00013 (R) | 6.10E-07 | 16 (3.1) |
| 20:34,918,046 C / G (rs117344844) | ACSS2 | 0.03 (C) | 6.40E-07 | 0.60 (0.12) |
| 19:47,181,548 G / A (rs149198684) | SAE1 | 0.049 (C) | 6.60E-07 | 0.47 (0.095) |
| 4:167,926,776 A / G (rs112517276) | ANXA10 | 0.00019 (R) | 6.60E-07 | 14 (2.7) |
| 1:169,135,186 C / CA | NME7 | 0.00024 (R) | 7.10E-07 | 11 (2.2) |
| 8:143,046,354 G / A (rs770749259) | LY6E | 0.00034 (R) | 7.10E-07 | 12 (2.4) |
| 5:79,217,485 G / A (rs963316704) | DMGDH | 0.000058 (R) | 7.80E-07 | 39 (8.0) |
| 11:105,056,466 G / A (rs117902177) | CARD16 | 0.015 (C) | 8.30E-07 | 0.87 (0.18) |
| 16:47,283,923 T / C | ITFG1 | 0.000087 (R) | 8.30E-07 | 23 (4.6) |
| 16:47,838,804 A / T | PHKB | 0.000087 (R) | 8.30E-07 | 23 (4.6) |
| 18:77,935,620 C / G (rs534458410) | GALR1 | 0.00013 (R) | 8.40E-07 | 21 (4.3) |
| 16:67,151,374 G / T (rs778784601) | B3GNT9 | 0.00033 (R) | 8.60E-07 | 9.0 (1.8) |
| 13:73,886,625 T / C (rs571359406) | KLF12 | 0.00017 (R) | 8.70E-07 | 14 (2.8) |
| 16:46,572,159 A / T (rs997757941) | SHCBP1 | 0.000087 (R) | 8.80E-07 | 23 (4.6) |
| 1:230,050,404 T / G (rs772149030) | GALNT2 | 0.0016 (C) | 9.70E-07 | 3.4 (0.70) |
| 3:154,890,200 A / G (rs548533017) | MME | 0.0027 (C) | 9.70E-07 | 2.4 (0.49) |
| 5:73,869,634 C / T (rs551854952) | ARHGEF28 | 0.000092 (R) | 9.80E-07 | 33 (6.7) |
| 4:172,842,621 G / A (rs17058962) | GALNTL6 | 0.00072 (R) | 9.90E-07 | 5.3 (1.1) |
| Gene variants found using the UKBiobank TOPMed-imputed PheWeb database. Diabetic retinopathy (ICD: 250.7). Variant classification, C = Common (MAF > 0.001), R = Rare (MAF < 0.001). | | | | |
